# Ketogenic diet improves the spatial memory impairment caused by exposure to hypobaric hypoxia through increased acetylation of histones in rats

**DOI:** 10.1371/journal.pone.0174477

**Published:** 2017-03-29

**Authors:** Ming Zhao, Xin Huang, Xiang Cheng, Xiao Lin, Tong Zhao, Liying Wu, Xiaodan Yu, Kuiwu Wu, Ming Fan, Lingling Zhu

**Affiliations:** 1 Department of Cognitive Science, Beijing Institute of Basic Medical Sciences, Beijing, P.R.China; 2 Co-innovation Center of Neuroregeneration, Nantong University, Nantong, JS, China; 3 Beijing Institute for Brain Disorders, 10 Xitoutiao, You Anmen, Fengtai District, Beijing, P. R. China; Bilkent University, TURKEY

## Abstract

Exposure to hypobaric hypoxia causes neuron cell damage, resulting in impaired cognitive function. Effective interventions to antagonize hypobaric hypoxia-induced memory impairment are in urgent need. Ketogenic diet (KD) has been successfully used to treat drug-resistant epilepsy and improves cognitive behaviors in epilepsy patients and other pathophysiological animal models. In the present study, we aimed to explore the potential beneficial effects of a KD on memory impairment caused by hypobaric hypoxia and the underlying possible mechanisms. We showed that the KD recipe used was ketogenic and increased plasma levels of ketone bodies, especially β-hydroxybutyrate. The results of the behavior tests showed that the KD did not affect general locomotor activity but obviously promoted spatial learning. Moreover, the KD significantly improved the spatial memory impairment caused by hypobaric hypoxia (simulated altitude of 6000 m, 24 h). In addition, the improving-effect of KD was mimicked by intraperitoneal injection of BHB. The western blot and immunohistochemistry results showed that KD treatment not only increased the acetylated levels of histone H3 and histone H4 compared to that of the control group but also antagonized the decrease in the acetylated histone H3 and H4 when exposed to hypobaric hypoxia. Furthermore, KD-hypoxia treatment also promoted PKA/CREB activation and BDNF protein expression compared to the effects of hypoxia alone. These results demonstrated that KD is a promising strategy to improve spatial memory impairment caused by hypobaric hypoxia, in which increased modification of histone acetylation plays an important role.

## Introduction

Hypobaric hypoxia is defined as a condition caused by the decreased partial pressure of oxygen in the atmosphere at high altitude, leading to reduced oxygen delivery to tissues. The brain is particularly sensitive to hypobaric hypoxia stress due to its high oxygen requirement. Moreover, in addition to structural alterations of the brain, high-altitude cerebral edema, neuroinflammation, motor impairment, memory impairment, etc. develop with exposure to hypobaric hypoxia [1, 2]. Regarding hypobaric hypoxia-induced memory impairment, an early report found that exposure to hypobaric hypoxia above 4700 m resulted in serious adverse changes in behavior performance, especially learning and memory [3]. Pagani et al. found that a significant impairment of spatial memory in mountaineers has been reported at altitudes greater than 5350 m for more than 15 days [4]. Based on animal models and biochemical screens, these cognitive deficits may be attributed to several molecular mechanisms, such as oxidative stress, glutamate-mediated excitotoxicity, altered ion channels, etc. Accordingly, antagonizing these signaling pathways plays a preventive role in ameliorating the impaired memory function [5]. Since hypobaric hypoxia exposure produces a complex and comprehensive stress on the brain, targeting a single pathway usually produces inadequate neuroprotection with limited efficacy. The development of new interventions is in urgent need.

Ketogenic diet (KD), a high-fat with low-carbohydrate diet, has been an effective treatment of intractable epilepsy in children for decades. Increasing evidence shows that KD also has a positive impact on cognition and behavior in various neurological disorders [6]. For example, Ijff et al. found that the KD exerts a positive impact on behavior and cognitive functioning in children and adolescents with refractory epilepsy [7]. In a rat epilepsy model kindled with pentylenetetrazol, KD not only attenuated the decrease in the exploration ratio in both the new object recognition and novel placement recognition task but also had a protective effect on spatial memory in the Morris water maze [8]. Although increasing evidence that the KD enhances cognitive function in both pathophysiological and normal healthy experimental animal systems [9,10], the exact mechanism of action by which the KD exerts these effects has not been fully understood. Previous studies have focused on its property of tissue energy transition and anti-oxidative damage. However, the recent findings that beta-hydroxybutyrate (BHB), the main ketone body of KD metabolism, is an endogenous and specific inhibitor of histone deacetylase (HDAC) provide new insights into the understanding of KD activity [11]. It is suggested that the cognition-improving effect of the KD might be related to increased histone acetylation modification.

In addition to activation of the Hypoxia-inducible factor-1 (HIF-1) pathway, hypoxia also provokes different epigenetic mechanisms involved in gene expression [12]. A recent report found that hypobaric hypoxia has differential effects on acetylation of proteins in the neocortex and hippocampus of the rat brain and that the predominant targets for this acetylation appear to be histones [13]. Reversing the decrease in histone acetylation may be an effective method to relieve injuries caused by hypobaric hypoxia. We proposed that KD may improve cognitive dysfunction caused by hypobaric hypoxia through modification of histone acetylation. In the present study, we found that KD administration not only enhances spatial learning in adult rats but also reverses the spatial memory impairment caused by hypobaric hypoxia. Moreover, the decrease in histone acetylation induced by hypoxia was reversed by KD treatment. These findings indicate that a KD can protect against memory impairment caused by exposure to hypobaric hypoxia.

## Materials and methods

### Ethics statement

All experimental procedures were approved by the Institutional Animal Care and Use Committee of Beijing Institute of Basic Medical Sciences and performed in accordance with the guidelines. During treatment, behavioral testing, and tissue collection procedures were devised to minimize the potential pain and distress of the animals used in this study. All rats were frequently monitored, at least three times a week, for health status.

### Animals and treatment

Eight-week-old male Sprague Dawley rats, purchased from Vital River Laboratory Animal Technology Co. (Beijing, China), were housed in a temperature- and humidity-controlled room. The rats were maintained under a 12-h light-dark cycle and given food and water *ad libitum*. Prior to the experiments, rats were allowed to acclimatize for three days. Rats were fasted overnight before being assigned into two groups fed with either the standard growing/maintenance diet (STD) or KD (purchased from and made by, respectively, by Beijing Vital River Laboratory Animal Technology Co.). The composition of the KD in a previous report was used here [14] and is shown in Table 1. Body weights were measured every three days until the end of the experiment. For hypobaric hypoxia treatment, rats were kept in a hypobaric hypoxia chamber (Guizhou Fenglei Aeronautics Ordnance Co. Ltd, China, model: DYC-DWI) for 24 h at a constant simulated altitude of 6000 m. Humidity in the chamber was maintained at 40–50% and the temperature at 22–24°C. Rats had free access to food and water and were maintained in a 12-h light-dark cycle. Rats were anaesthetized by intraperitoneal injection of pentobarbital sodium (2%, 1 ml/rat) and sacrificed by decapitation.

**Table 1:** Composition of STD and KD(g/100g).

Components	STD	KD
**Fat**	**5.28**	72*
**Protein (casein)**	**22.1**	**16**
**Carbohydrates (sucrose)**	**52**	**2**
**AIN-76 mineral mix**	**2.16**	**3.5**
**AIN-76 Vitamin mix**	**1**	**1**
**Cellulose**	**4.12**	**5**
**Calorie density (kcal/g)**	**3.5**	**7.2**
**Ketogenic ratio**	**0.07:1**	**4:1**

*: containing lard 44%, butter 17%, soybean oil 10%, sunflower oil 1%.

Abbreviations: STD, standard growing/maintenance diet; KD, ketogenic diet

### Biochemical analysis and tissue preparation

For biochemical analysis of the blood, samples were collected from tail vein blood and were transferred into EDTA-containing tubes. After centrifugation at 1500×*g* for 15 min at room temperature, the plasma was transferred into a new tube and stored at -80°C until use. The plasma levels of total cholesterol and triglyceride were measured using an ELISA kit (E1005 and E1003) from Applygen Technologies Inc. (Beijing, China). The plasma levels of BHB and acetoacetate (AcAc) were measured using an ELISA Kit (ab83390 and ab180875) purchased from Abcam (Cambridge, MA). Assays were performed according to the protocols provided by the manufacturer. After behavioral tests, the rats were anaesthetized and sacrificed by decapitation. The brains were rapidly removed, and the hippocampus was dissected and frozen at -80°C for future western blot analysis. The brains used for immunohistochemistry analysis were fixed in 4% formaldehyde immediately after isolation.

### Open field test

Rats were placed in a square open field chamber (100×100×40 cm, length × width × height) with a smooth black floor and black walls. The floor was divided into 25 squares of 20×20 cm. Rat were put in the center of the chamber and were allowed to explore freely for five min. Their behavior was recorded by a digital camera located above the chamber. Using ANY-maze video imaging software (Stoelting Co., IL), the time spent in the center area as well as the number of entries into the center area (inner 9 of 25 squares) were determined. Locomotor behavior was calculated as the distance traveled during the test. The area was wiped clean with 70% ethanol between each test.

### Morris water maze test

The water maze apparatus was a circular pool (diameter = 160 cm, height = 50 cm) filled with water (temperature approximately 22–24°C). An invisible styrofoam platform was placed 1 cm below the water surface. Four visual cues were placed on the curtain (above the pool edge) that surrounded the maze. The pool was divided into four quadrants. Behavior was monitored and recorded by a digital camera interfaced to a computer installed with the ANY-maze video imaging software (Stoelting Co., IL). During the five days of the acquisition training period, the rats were placed in the water facing the midpoint section of each of the quadrants. Rats were allowed to swim freely until they found and climbed onto the platform. If a rat failed to locate the platform within 90 s, it was placed on the platform for 10 s. Each rat was subjected to 4 trials per day, and the starting position was changed each day. For the spatial probe test, the platform was removed and the rat was placed into the water at a quadrant. The number of times the rat passed the previous location of the platform, the time in the target quadrant, and the latency to first pass the platform location within 90 s were recorded.

### Western blot analysis

Frozen hippocampi were disaggregated in ice-cold RIPA buffer containing protein inhibitors. The dissolved proteins were collected after centrifugation at 10,000×g for 10 min at 4°C, and the supernatant was then collected. The protein concentration was determined using a Pierce BCA Protein Assay kit (Thermo Scientific). A quantity of 50 μg of total protein was loaded onto a 10–15% SDS-PAGE, electrophoretically transferred to polyvinylidene difluoride membrane (PVDF), and probed with the following primary antibodies: Acetyl-Histone H3 (06–599, Merck Millipore), Acetyl-Histone H3 (Lys14) (07–353, Merck Millipore), Histone H4 (Acetyl K12) (ab61238, Abcam), Histone H3 (4499, Cell Signaling Tech.), Histone H4 (2935, Cell Signaling Tech.), phospho-CREB (Ser133) (9198, Cell Signaling Tech.), CREB (4820, Cell Signaling Tech.), phospho-(Ser/Thr) PKA substrates (9621, Cell Signaling Tech). β-actin (A2228, Sigma-Aldrich) was used as an internal control. The membranes were developed using an enhanced chemiluminescence detection system (Pierce, Rockford, IL). For densitometry of the protein bands, the optical densities (OD) of the protein bands were quantified using Quantity One software (Bio-Rad). The relative band densities were calculated as the ratio of the protein band OD relative to the β-actin (internal loading control) band OD from the same sample. The ratios for the untreated control samples were set to 1.

### Immunohistochemistry (IHC) staining

For immunohistochemistry analysis, the fixed brains were then embedded into paraffin according to a standard histological protocol and sectioned. The coronal sections (5 μm) of the brain were incubated overnight with the antibodies against Acetyl-Histone H3 (dilution 1:100) and Histone H4 (dilution 1:50). Following incubation with biotinylated goat anti-mouse antibody, the signals were visualized using the ABC kit (Vector Lab, Burlingame, CA).

### Quantitative real-time PCR (qRT-PCR)

Total RNA was extracted from the hippocampus of the mouse brains using Trizol reagent (Invitrogen, USA). First-stand cDNA of each sample was synthesized using an MLV reverse transcription kit (TAKARA, Japan) according to the manufacturer’s instructions. cDNA was used as a template for quantitative real-time PCR using SYBR green master mix (Applied Biosystems, USA). The primers used are as follows: BDNF-F: 5ˊ- TGGCTGACACTTTTGAGCAC-3ˊ, BDNF-R: 5ˊ-GAAGTGTACAAGTCCGCGTC-3ˊ; β-actin-F: 5ˊ-TTGCCCTAGACTTCGAGCAA-3ˊ, β-actin-R: 5ˊ- CAGGAAGGAAGGCTGGAAGA-3ˊ. Gene expression was normalized to the mRNA levels of β-actin.

### Chromatin immunoprecipitation (ChIP)

ChIP was performed following a minor modification of the EZ-ChIP kit protocol (Merck Millipore). Briefly, hippocampus was minced in ice-cold PBS and cross-linked with 1% formaldehyde at room temperature for 15 min. Cross-linking was stopped by addition of glycine to a final concentration 0.125M. Sample were washed in ice-cold PBS containing protease inhibitors (Roche Diagnostics), and stored at -80°C. The fixed tissue was resuspended in SDS lysis buffer (Merck Millipore) containing protease inhibitors and 1 mM PMSF. Each sample was sonicated to generate chromatin fragments with an average length of 200–500 bp. The sample was centrifuged at 12 000 g at 4°C for 15 min, and the supernatant chromatin was saved and protein concentration was estimated by BCA method. The sample was further diluted in dilution buffer. The suspension was incubated with Protein A-sepharose bead slurry for 2 h at 4°C and then centrifuged at 3500 g at 4°C for 10 min. The supernatant was saved and divided into input and immunoprecipitation. Acetyl-Histone H3 (06–599, Merck Millipore) antibody (4 μg) was added to immunoprecipitation fraction and incubated overnight at 4°C. Next day, Protein A-sepharose bead slurry (50 μL) was added to it and rotated for 2 h at 4°C and then centrifuged at 3500 g for 10 min at 4°C. The pellet was washed in low salt, high salt, LiCl, and TE buffer. The immune complex was eluted by elution buffer. For reverse cross-linking of the protein–DNA complex, 200 mM NaCl was added to the pellet, and followed by RNase A and proteinase K digestion. DNA was extracted by column purification kit provided by the kit, and resuspended in 10 mM Tris pH8.0

The sequences of interest were amplified by qRT-PCR method using specific primers for BDNF promoter I (pI) (pI-F: 5ˊ- GCAGTTGGACAGTCATTGGTAACC -3ˊ; pI-R: 5ˊ- ACGCAAACGCCCTCATTCTG -3ˊ) [15]. PCR reactions were performed in triplicate. Threshold amplification cycle numbers (C_t_) were used to calculate IP DNA quantities as % of input controls.

### Statistical analysis

Statistical analysis was performed using GraphPad Prism 5 (GraphPad Software Inc., San Diego, CA), and the data are expressed as the mean ± SEM. For data with equal variances, a one-way analysis of variance (ANOVA) followed by Dunnett's test was performed.

## Results

### KD increases the plasma levels of lipids and ketone bodies

**Fig 1:**
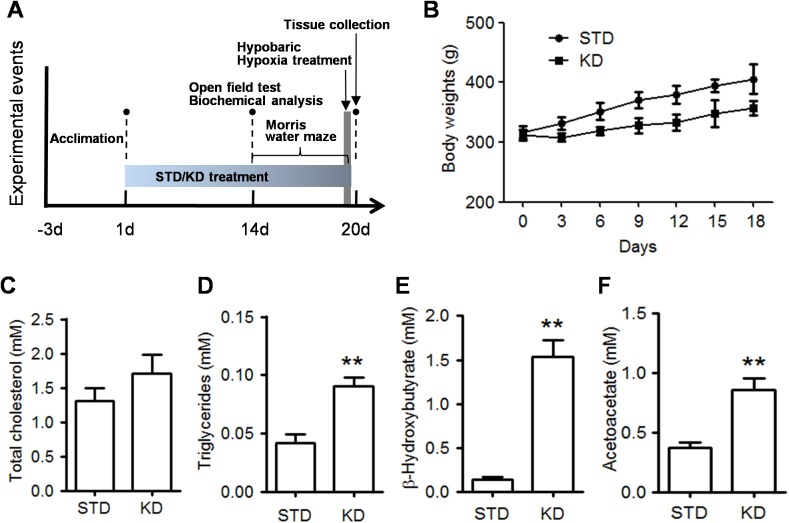
The effect of KD treatment on plasma levels of lipids and ketones. **A**. Schematic of the experimental design. Rats were acclimated for 3 days and were then randomly divided into two groups (n = 20 each) fed with either the standard diet (STD) or ketogenic diet (KD). After 14 days of treatment, the rats were subjected to an open field test and biochemical analysis. Next, the rats were subjected to the Morris water maze test for 6 days and hypobaric hypoxia treatment (10 rats in each treatment group) between the 5th day of acquisition training and the probe test on the 6^th^ day. B. The rats body weights were recorded during whole experimental period. Data are presented as mean ± S.E. **C-F**. The plasma levels of total cholesterol (C), triglycerides (D), BHB (E), and AcAc (F) were detected by ELISA assay. Five rats in each group. Values are presented as the mean ± S.E. (**p<0.01).

The experiments were designed as shown in Fig 1A. After three days of acclimation, the rats were fed with the STD or KD for two weeks and were then subjected to biochemical analysis and behavioral tests. First, the effects of the two diet on body weight were recorded every three days, and we found that rats fed on the KD had a slower weight gain compared with rats in STD group (Fig 1B). We then detected the effect of KD administration on the lipid metabolism. As shown in Fig 1C & 1D, the total cholesterol in the KD rats was slightly higher than that in the STD rats, but there was no significant difference between the two groups. In contrast, the triglyceride levels in the KD rats were much higher than that in the STD rats (p<0.01). We next measured the plasma levels of ketone bodies, which are metabolized from fatty acid oxidation. We found that the plasma levels of BHB and AcAc were all increased in the KD rats compared to that in the STD rats (Fig 1E & 1F). In particular, the BHB concentration reached a mean value of 1.53 mM in the KD compared to 0.14 mM in the STD group. These results demonstrated that the KD recipe used in our study was ketogenic.

### KD does not affect locomotor activity but enhances spatial learning in adult rats

**Fig 2:**
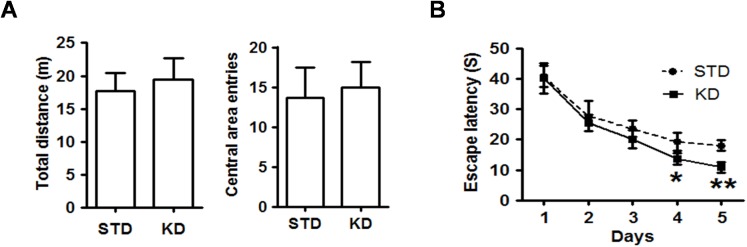
KD treatment enhances spatial learning and memory in adult rats. **A**. The total distance travelled and the number of entries into the central area in the open field test were evaluated. Values are presented as the mean ± S.E. (n = 10). **B** The mean of the escape latencies to find the hidden platform across the four trials are shown for the five days during the acquisition training period. Ten rats in each group.

The open field test was used as a standard test of general activity. We examined the effect of 2 weeks of KD administration on the general locomotor activity. As shown in Fig 2A, there was no difference in the total distance travelled and number of entries into the center area between the rats in the two groups, indicating that KD treatment did not affect the general locomotor and emotional activity. To detect the effect of KD on spatial learning and memory, the rats were subjected to the Morris water maze. During the five days of acquisition training, the escape latency of the rats in each group decreased day by day, but the average latency of the KD rats was less than that of the STD rats began on the fourth day (Fig 2B), indicating that KD treatment enhanced spatial learning ability.

### KD improves hypobaric hypoxia-induced memory impairment

**Fig 3:**
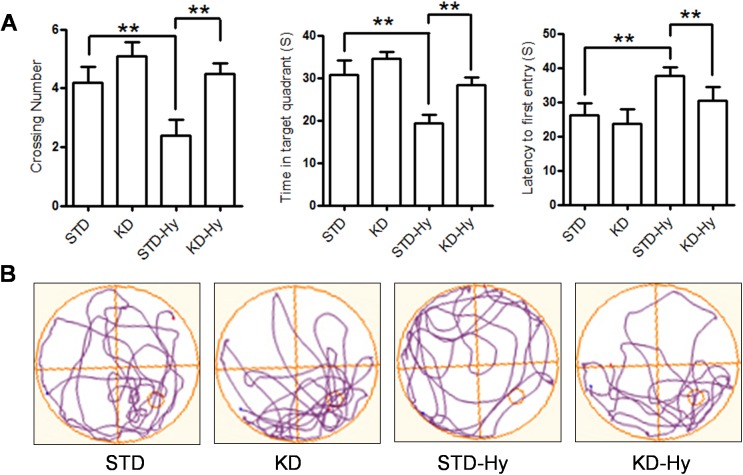
KD treatment reversed the spatial impairment induced by exposure to hypobaric hypoxia. **A**. The mean number of platform crossings, the mean time in the target quadrant, and the latency to first pass the location of the original platform during the probe test were evaluated. Values are presented as the mean ± S.E. (n = 10). **B**. Representative locomotion tracking plots showing the total path-length during the probe test. The red circle indicates the platform location.

To investigate whether KD treatment can improve memory impairment caused by hypobaric hypoxia, the rats were moved into a hypoxia chamber (simulated high altitude of 6000 m) prior to the spatial probe test. After 24 h of exposure, the rats were subjected to a probe test. The results showed that exposure to hypoxia resulted in obvious memory impairment, indicated by fewer crossings and less time and distance spent in the target quadrant than that of the control group. However, all of these parameters were reversed by KD treatment (Fig 3A). Compared to rats in the hypobaric hypoxia group, the rats in the KD-hypoxia group exhibited enhanced preference for the target quadrant over other quadrants (Fig 3B). These results showed that KD exhibits an evident beneficial effect on hypobaric hypoxia-induced memory impairment.

### Exogenous BHB prevents spatial memory impairment induced by hypobaric hypoxia

**Fig 4:**
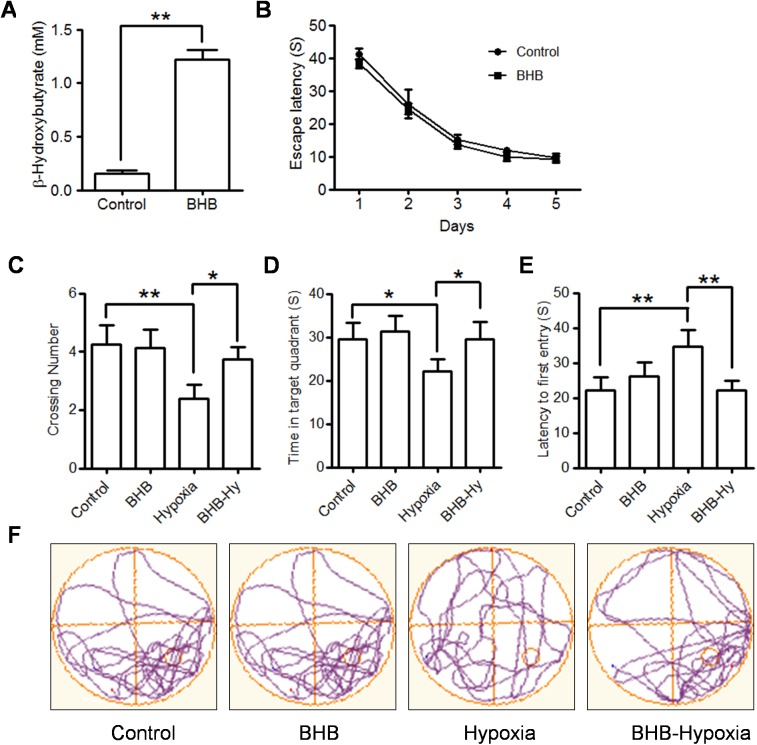
Exogenous BHB has direct improving-effects on spatial impairment induced by hypobaric hypoxia. **A**. The plasma levels of BHB were detected by ELISA assay. Five rats in each group. Values are presented as the mean ± S.E. (**p<0.01). **B** The mean of the escape latencies to find hidden platform across the four trials are shown for the five days during the acquisition training period. Ten rats in each group. **C-E**. The mean number of platform crossings (C), the mean time in the target quadrant (D), and the latency to first pass the location of the original platform (E) during the probe test were evaluated. Values are presented as the mean ± S.E. (n = 10, *p<0.05, **p<0.01)). **F**. Representative locomotion tracking plots showing the total path-length during the probe test. The red circle indicates the platform location.

To further verify whether ketone body, a product of KD, has direct improving effect, we chose the most stable physiologic ketone body, BHB, for the subsequent experiment. In order to mimic the effect of KD as above described, the rats were pre-treated with BHB (at a dose of 200mg/kg/day) for 2 weeks and then submitted to Morris water maze test. Since intraperitoneal injection would allow substances to be absorbed at a slower rate and intraperitoneal injection would produce marginal effect during behavioral tests [16], we used the intraperitoneal injection of BHB, which has been applied in published reports [17, 18]. Although the rats in the control and BHB groups learned to find the platform with the same pattern during 5 days of acquisition training (Fig 4B), BHB could significantly improve the memory impairment induced by hypobaric hypoxia, represented by more crossing number, more time in the target quadrant, and decreased latency to first entry to platform compared to hypobaric hypoxia treatment alone (Fig 4C–4F). These results demonstrated that BHB has a direct memory-improving effect and served as the main executor of KD beneficial effects.

### KD increases histone acetylation modification in the hippocampus

**Fig 5:**
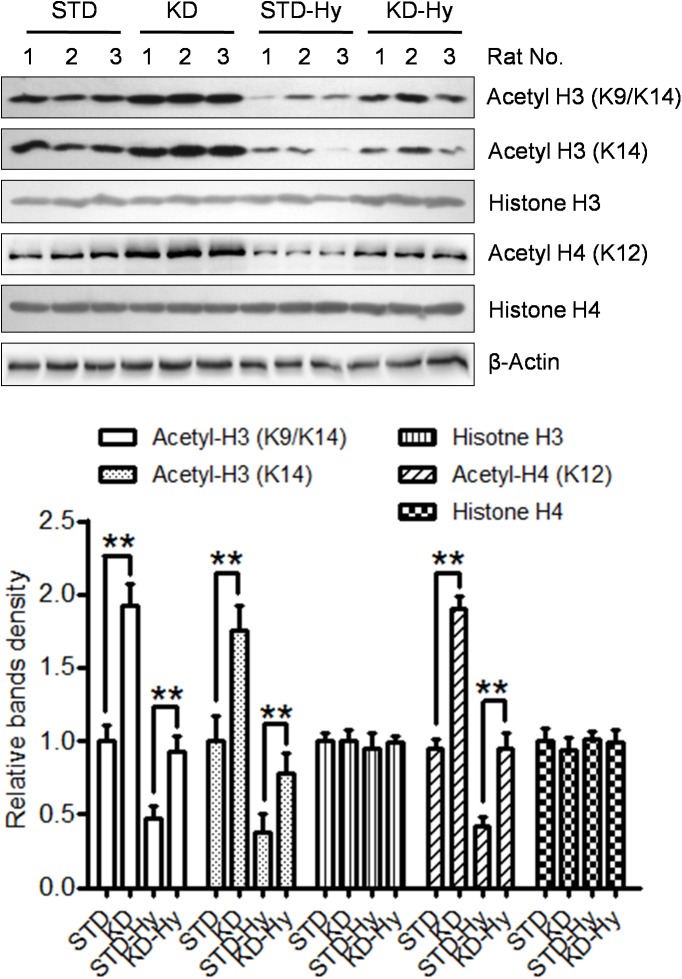
KD treatment increases histone acetylation in the hippocampus. Protein levels of acetyl-histone H3 (K9/K14), acetyl-histone H3 (K14) and acetyl-histone H4 (K12) in the hippocampus were detected by western blot. The levels of β-actin serve as an inner control. Three rats in each group. The right bar graph shows the relative band density of each protein in each group, and the data are shown as the means ± S.E. (** p<0.01).

**Fig 6:**
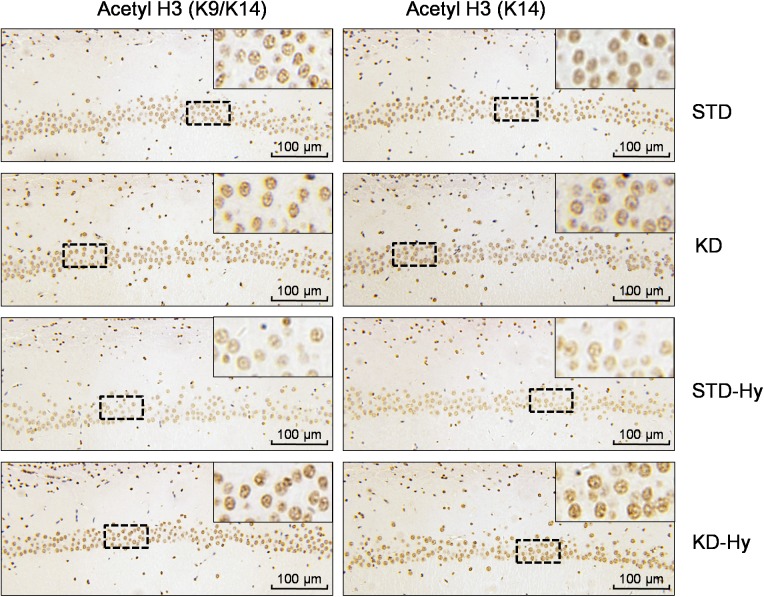
KD treatment increases histone acetylation in the CA1 region of the hippocampus. Representative images of immunohistochemistry analysis of acetyl-histone H3 (K9/K14) and acetyl-histone H4 (K12) in the CA1 region of the hippocampus. The insets show higher magnification of representative areas located in the dotted box.

A previous study found that BHB is an endogenous HDAC inhibitor, and the KD recipe in our study substantially increased plasma levels of BHB. Then, we detected the effect of KD on histone acetylation in the hippocampus, which is responsible for learning and memory. As shown in Fig 5, the acetylated histone H3 (K9/K14), acetylated histone H3 (K14), and acetylated histone H4 (K12), were all increased in the hippocampus of the KD rats. Although the histone acetylation modifications listed above are decreased in hypoxia-treated rats, KD treatment could reverse the decreased levels of histone acetylation. The same pattern was displayed in the immunohistochemical staining, in which the hypoxia-induced decrease in acetylated histone H3 and acetylated histone H4 in the CA1 region of the hippocampus was reversed by KD treatment (Fig 6).

### KD activates PKA/CREB signaling in the hippocampus

**Fig 7:**
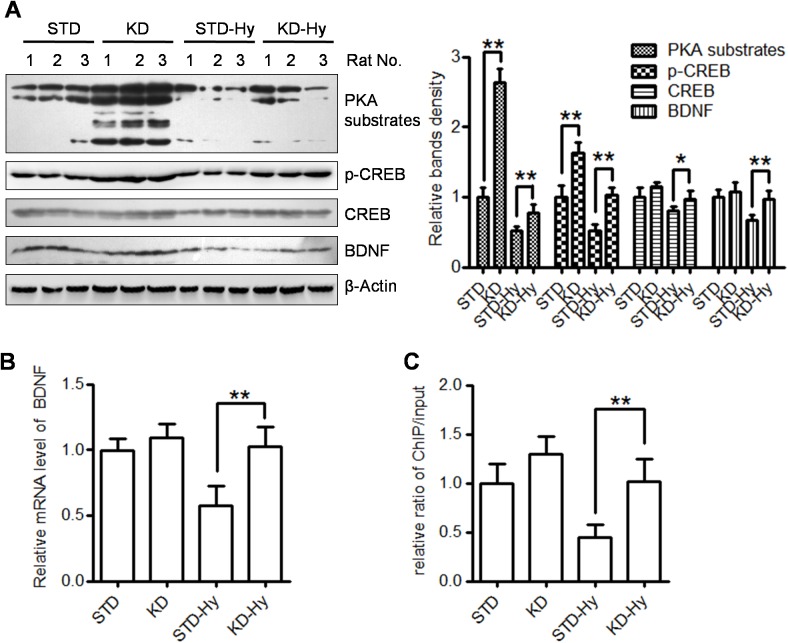
KD treatment promotes the PKA/CREB pathway and increase BDNF protein levels. **A**. Protein levels of PKA substrates, p-CREB, CREB and BDNF, in the hippocampus were detected by western blot. The levels of β-actin serve as an inner control. Three rats in each group. The right bar graph shows the relative band density of each protein in each group, and the data are shown as the means ± S.E. (* p<0.05, ** p<0.01). **B**. Quantitative realtime PCR of BDNF gene expression in the four groups. Data are expressed as means ± S.E, and normalized to the STD group (** p<0.01). **C**. ChIP-PCR were performed to measured the binding of acetylated histone H3 (Lys9/14) to the promoter I of BDNF gene. Threshold amplification cycle numbers (C_t_) were used to calculate IP DNA quantities as % of input control. Data are expressed as means ± S.E, and normalized to the STD group (** p<0.01).

To explore a possible underlying mechanism of the beneficial effect of KD treatment on cognition, the activity of the PKA/CREB pathway in the four groups was also evaluated by western blot (Fig 7A). KD treatment was shown to not only increase the levels of PKA substrates and p-CREB (KD *vs* STD) but also reverse the decline in PKA substrates, p-CREB and CREB (KD-Hy *vs* STD-Hy). Although KD pre-treatment produced a partial restoration of PKA activity, p-CREB is nearly completely restore to its basic levels, which is may be account for its other upstream kinases, like calmodulin-dependent kinases [19]. Interestingly, the hypoxia-induced down-regulation of BDNF, a well-known neurotrophic factor involved in learning and memory formation processes, was also up-reregulated by KD treatment. These results demonstrated that KD treatment promoted PKA/CREB activation and BDNF protein expression. In order to detect whether KD promoted BDNF expression at mRNA levels, qRT-PCR assays were performed using BDNF specific primers. We found that KD-pretreatment significantly increased mRNA levels compared with that in hypobaric hypoxia group (Fig 7B). Next, we used ChIP-PCR to test if there might be increased enrichment of acetylated histones on the promoter of BDNF gene. We focused on the promoter I of BDNF gene, which response to neuronal activity [20]. The results showed that there is increased binding of acetylated histone H3 to the promoter I of BDNF gene (Fig 7C).

## Discussion

In the present study, we found that KD treatment not only significantly enhanced spatial learning and memory in adult rats but also reversed the spatial memory impairment induced by hypobaric hypoxia. At the molecular level, we found that acetylated levels of histones, such as acetyl-histone H3 (K9/K14), acetyl-histone H3 (K14), and acetyl-histone H4 (K12), were increased by KD treatment. Moreover, KD treatment also reversed the decrease in histone acetylation caused by hypobaric hypoxia. These results demonstrated that KD might be a potential effective strategy to reverse the spatial memory impairment caused by exposure to hypobaric hypoxia, in which increased histone acetylation modification plays an important role.

The main ketone body produced in the liver is AcAc, but the primary circulation ketone is BHB. Thus, we first confirmed that the KD regime used in our study is truly ketogenic (Fig 1D & 1E). The plasma levels of BHB increased to 1.53 mM. Recent studies have demonstrated that BHB is more than just a metabolite; it has important cellular signaling roles as well. The most intriguing finding is that BHB is an endogenous inhibitor of HDAC and regulates gene expression via chromatin modifications. A previous study found that BHB at 1–2 mM serum concentrations, which can be reached by prolonged fasting or calorie restriction, can result in significant HDAC inhibition and induces specific gene expression [11]. In the present study, the western blot results from the hippocampus tissue showed that KD moderately increase the acetylated levels of histone, such as acetylated histone H3 (K9/K14, K14) and acetylated histone H4 (K12). Moreover, KD treatment exhibited a reversal effect on the decreased histone acetylation caused by hypobaric hypoxia. The same pattern was displayed in IHC staining of the CA1 region. These results strongly support the hypothesis that BHB-mediated acetylation modification plays a crucial role in the memory-improving effect induced by KD. This notion was also supported by clinical trials involving patients with AD or mild cognitive impairment [21]. An orally administered mixture of medium chain triglycerides was reported to improve memory and attention in these individuals, and better performance was associated with higher plasma BHB levels.

BDNF is considered as the most prevalent neurotrophic factor in the brain, responsible for neuronal survival and synaptic plasticity *et al*. Acute and chronic hypoxia have been reported to decrease the levels of BDNF in prefrontal cortex and hippocampus. Anti-hypoxia treatment can increases the expression of BDNF in these regions [22, 23]. Activation of the BDNF signaling pathway may be involved in the hypoxia-insult protection. More importantly, it is also reported that the decreased BDNF may be involved in cognitive impairment during acute high altitude hypoxia in human volunteers [24]. In the present study, we found that the decreased levels of BDNF protein are restored by KD pre-treatment, which is accompanied by increased levels of acetylated histone H3 and H4 in the hippocampus. Moreover, The results of ChIP-PCR showed that there is increased acetylated histone H3 binding to promoter I of BDNF gene. Since KD is ketogenic and BHB is known serves as HDACs inhibitor, BHB is probably the executor via its HDAC inhibiting activity to specifically induce BDNF expression. This propose is efficiently supported by a newly published report that both exercise-induced BHB and exogenous injected BHB specifically increases BDNF promoter I activity in the hippocampus of mice, which is accompanied by inhibition of HDAC2 and HDAC3 activity [25]. Therefore, KD restores memory-related gene expression through the enhancement of histone acetylation at gene promoters.

It is well known that epigenetics, especially histone acetylation, play crucial roles in the cellular response to hypoxia. Histone acetylation is necessary for the protection of brain function from injury through remodeling of chromatin structure and, consequently, gene expression [26]. The results of the western blot in our study showed that the reversal of the decreased histone acetylation provided a foundation for KD treatment to protect the brain from hypoxia-induced memory impairment. This notion is also supported by a new published report that severe hypobaric hypoxia (equivalent to an altitude of 11 km) caused global repression of the acetylation process in the neocortex and hippocampus in 3–24 h [13]. It has also been found that up-regulation of histone acetylation by HDAC inhibitor treatment improved long term memory performances in several hippocampal-dependent paradigms, such as contextual fear conditioning [27], Morris water maze [28], and object location [29]. Moreover, in addition to rescuing cognitive impairment resulting from neurodevelopmental and neurodegenerative disorders, HDAC inhibitors also have great potential to serve as cognitive enhancers for the cognitively healthy. Administration of the HDAC inhibitor SAHA or NaB resulted in enhanced contextual fear memory performance in mice and improved spatial memory on the water maze task, respectively, [30]. Therefore, targeting dysregulated histone acetylation is a promising strategy to modulate memory functions in various neural disorders, including hypobaric hypoxia-induced memory impairment.

In combination with previous findings, our data provide new evidence that there is a promising application for the KD in improving cognition of neural disorders resulted from hypoxia injury. Evidence supporting this proposal comes from findings that ketosis, whether induced by a KD or by administration of BHB, plays a protecting role in various models. For example, administration of 4 mM Na-BHB, a concentration observed in mild ketosis, has been reported to enhance the survival of primary cultured hippocampal neurons exposed to hypoxia in serum-free media by decreasing apoptotic damage [31]. In the organotypic hippocampal culture model, exogenous ketone administration of BHB not only preserved neuronal integrity and stability but also protected cells from the metabolic and excitotoxic insults and reduced post-hypoxic hyperexcitability [32,33]. Oral KD or intraventricular-infused BHB also resulted in a significant reduction in infarct volume following MCAO in rats [34]. Taken together, these data suggest that a KD might be a strong candidate for treatment of neural disorders resulting from hypoxia injury. Since the clinical safety of a KD has already been verified, it would be interesting to determine the preventive effects of a KD on high mountain disease in volunteers at a high-altitude plateau.

Although more studies are necessary to fully define the fundamental mechanisms of KD-induced cognitive enhancement, the present data may offer valuable clues towards a better understanding of a KD in the improvement of cognitive disorder. Our results also lay the foundation for future research by identifying the critical improvement in synaptic plasticity, the molecular basis of cognitive flexibility. As our knowledge of KD as an effective intervention for cognitive disorders continues to expand, future research aimed at exploring the impact of a KD on cognition deterioration when reaching a high-altitude plateau is of great clinical significance.
